# Natural environments, ancestral diets, and microbial ecology: is there a modern “paleo-deficit disorder”? Part II

**DOI:** 10.1186/s40101-014-0040-4

**Published:** 2015-03-10

**Authors:** Alan C Logan, Martin A Katzman, Vicent Balanzá-Martínez

**Affiliations:** CAMNR, 23679 Calabasas Road Suite 542, Calabasas, CA 91302 USA; Department of Psychiatry, Faculty of Medicine, University of Toronto, 250 College Street, 8th floor, Toronto, ON M5T 1R8 Canada; Section of Psychiatry, Department of Medicine, University of Valencia Medical School, Avda. Blasco Ibáñez, 15. E46010, Valencia, Spain

## Abstract

Famed microbiologist René J. Dubos (1901–1982) was an early pioneer in the developmental origins of health and disease (DOHaD) construct. In the 1960s, he conducted groundbreaking research concerning the ways in which early-life experience with nutrition, microbiota, stress, and other environmental variables could influence later-life health outcomes. He recognized the co-evolutionary relationship between microbiota and the human host. Almost 2 decades before the hygiene hypothesis, he suggested that children in developed nations were becoming too sanitized (vs. our ancestral past) and that scientists should determine whether the childhood environment should be “dirtied up in a controlled manner.” He also argued that oft-celebrated growth chart increases via changes in the global food supply and dietary patterns should not be equated to quality of life and mental health. Here in the second part of our review, we reflect the words of Dubos off contemporary research findings in the areas of diet, the gut-brain-axis (microbiota and anxiety and depression) and microbial ecology. Finally, we argue, as Dubos did 40 years ago, that researchers should more closely examine the relevancy of silo-sequestered, reductionist findings in the larger picture of human quality of life. In the context of global climate change and the epidemiological transition, an allergy epidemic and psychosocial stress, our review suggests that discussions of natural environments, urbanization, biodiversity, microbiota, nutrition, and mental health, are often one in the same.

“*But it can be surmised that the consumption of new kinds of food will bring in its train new types of medical problems. Nutritional disease can arise not only from qualitative or quantitative deficiencies, but also from toxic effects, which are often slow in manifesting themselves*…*scientific knowledge is not yet sufficient to replace the biological wisdom derived from the countless centuries during which mankind has engaged in the empirical trial of foodstuffs*” [1].

“*In my judgment we are about to enter a different phase of nutritional sciences. One based on functional tests determining the role of food not only in growth and disease, but in the various functions which enable the body and mind to respond effectively to challenges and stresses in the various manifestations of life…when, and if, we reach the proper level of knowledge, nutrition will become part of a new science, as yet undeveloped, human ecology*” [2].

Outside of his focus on the relationship between nutrition and intestinal microbiota, which we will discuss later, Dubos did not write extensively on nutritional matters. Still, as evidenced from the quotes above, his broad view of nutrition was through the same ecological and ancestral lens with which he surveyed other topics. However, and this is a salient point, he did not advocate for some type of universal Stone Age cuisine. He scoffed at such notions, stating that “*unfortunately for clinical nutritionists (but fortunately for the human species), people can remain healthy and can function effectively on a great diversity of dietary regimens that are far different from those considered scientifically ideal*” [3].

Modern research has certainly supported his position. Depending on their residential environment, our hunter-gatherer ancestors sustained themselves using an enormous variety of foods [4-6]. Indeed, the remarkable dietary flexibility exhibited by our ancestors seems responsible for the success and expansion of the genus *Homo* [7]. Interestingly, the modernized global food supply pushes homogeneity such that the diversity of crops contributing to worldwide nutrition, including starchy roots, has declined [8]. Regardless of the cartoonish “rack of ribs” stereotypes, ancestral diets are united in what they included (plants) and what they did not include (ultra-processed foods).

Food consumption and household food expenditure trends in Canada (1938–2011) present a clear picture of the dietary shift in the last century. Unprocessed or minimally processed roots and tubers as a contribution to household caloric intake have declined by 80%. On the other hand, the consumption of ready-to-consume processed and ultra-processed foods has more than doubled. There has been a 32% increase in household food budget share devoted to ready-to-eat processed and ultra-processed foods. The largest jumps have been in processed meats, sweetened beverages, spreads and sauces (including mayonnaise- and margarine-containing products), and sweetened baked goods [9]. At the same time, the latest research shows that Canadian adults (age 19–50) consume only 0.5 servings of dark green vegetables and 74% of the population aged 2 and older were *not* meeting Health Canada’s guidelines for fruit and vegetable intake [10].

During his time of writing, “proper nutrition” was being celebrated for causing rapid gains in height and weight of populations such as the Japanese. Dubos discussed the associations between a 15-fold increase in milk consumption (plus 7.5-fold increase in egg and meat consumption—1950–1975) and marked Japanese growth chart increases; however, he did not equate increasing height with quality of life. As he said, “*the post-war Japanese are taller than their parents, but this does not mean they will live longer, will be happier or will become more productive in the arts and sciences*” [2].

He urged scientists to examine the behavioral aspects of nutrition, to more closely evaluate the ways in which nutrition allows an organism to make adequate “*biological and psychological responses to various life situations*” [2]. He encouraged the use of objective markers wherever possible. Moreover, Dubos argued for research into the ways in which psychological distress and disturbances in circadian rhythms can influence the metabolic demands for, and fate of, various nutrients [3]—topics that only recently have become the subject of scientific scrutiny [11,12].

Adherence to ancestral dietary patterns, exemplified by the Mediterranean or Paleolithic descriptive, has been linked with many favorable health outcomes [13-15]. Detailed analysis of the mid-Victorian period reveals that for a very brief period in United Kingdom history, an ideal combination of the best of ancestral diets and high levels of physical activity culminated in remarkable life expectancy (beyond infancy) and absence of degenerative disease [16]. However, by 1880, the era of processed, high-sugar, and low-nutrient foods began.

Recent advances in the field of nutritional psychiatry have made it clear that there are indeed remarkable ways in which nutrition influences brain structure and function, as well as mental health and cognition [11,17]. Even short-term adoption of traditional dietary patterns can beneficially influence mood and cognition [18]. An increasing number of epidemiological studies, including several that have followed subjects over time, have linked adherence to traditional dietary patterns with lowered risk of depressive symptoms, anxiety, and maintenance of academic progress [19-25]. Meat consumption, often painted with the same brush (without considering processing) and vilified without scientific justification [26,27], has been associated with lower risk of depression [28,29], and meat abstinence has been linked with higher risk of mental health disorder [30]. This is not to suggest that meat is a mental health panacea; a strict plant-based diet, lower in sweets and rich in fruits and vegetables, may also support positive mental health [31].

Connections between nutrition and mental health extend to mortality. For example, those with a lifetime history of attempted suicide have been reported to consume significantly less meat, fruits, and vegetables [32]. On the other hand, healthy dietary patterns characterized by higher intake of vegetables, fruits, potatoes, soy products, mushrooms, seaweed, and fish are associated with a decreased risk of suicide [33]. The developmental origins of health and disease (DOHaD) construct provides clear evidence that early life is a critical time in subsequent risk of non-communicable diseases [34,35]. With his groundbreaking studies on the long-lasting effects of environmental variables experienced in early life, Dubos helped to place DOHaD under the scientific microscope [36]. Today, pre- and post-natal nutrition is now being clearly linked to later mental health outcomes and childhood nutrition with academic performance [37-39].

Specific components of traditional dietary patterns, including, but not limited to cocoa polyphenols, green tea, coffee, grapes, and various spices, have also been linked to positive mood, cognitive efficiency, and a decreased risk of depressive symptoms [40-46]. Experimental research shows that the phytochemicals found within traditional foods (e.g., tea polyphenols and resveratrol) and omega-3 fats that are relatively more abundant in fish and free-range meats can influence brain function via neurotransmitter availability for synaptic communication [47-49]. They can also increase the production of neurotrophic factors responsible for neuronal structure and function [50-52]. The evolutionary advantage of phytochemicals to both plants and humans has been postulated [53].

When humans encounter psychological stress, they often turn to calorie-dense, nutritionally poor “comfort foods” [54-56]. In otherwise healthy adults, wherein negative mood state is experimentally induced by researchers, the direct infusion of fatty acids in the stomach (therefore bypassing olfactory, visual, and gustatory cues) can quickly rectify the lowered mood state [57]. As much as phytochemicals may protect us, are there evolutionary factors that might lead modern humans *away* from traditional dietary practices? Does fast food create a dependence? It would make sense that in our Paleolithic past, heightened pleasure associated with energy-dense food consumption would further motivate intake—a critical motivation given the frequency with which foods could become scarce.

The preponderance of epidemiological and experimental research indicates that the highly palatable addition or relative magnification of sugar, fat, and/or sodium contributes to the attractiveness of the contemporary ultra-processed diet [58-62]. Animal models of early-life stress demonstrate that the Westernized diet can minimize the physiological stress response, supporting the notion that consumption of palatable foods is a form of “self-medication” [63]. Moreover, the perinatal period represents a highly sensitive period in which dietary experience may dictate subsequent food preferences and mental outlook over the offspring life course [64,65]. For example, perinatal experience with a high-fat diet in animals increases the likelihood of anxiety behavior and the expression of corticosterone receptors in the amygdala in adulthood [66]. Taken together, the physiological responses to the consumption of energy-dense comfort foods are likely to be behaviorally reinforced in the contemporary environment wherein psychological distress and cognitive load are high.

Although tempting to dismiss the mental health value of traditional diets as largely unrelated to those of natural environments, there are multiple points at which the discussions become potentially one in the same. Closer residential proximity to urban green space is associated with healthier dietary habits and lower insulin resistance [67,68]. In traditional communities, such as in Malawi, the loss of forest cover is associated with diminished dietary diversity and an increased risk of nutritional gaps [69].

At the top level, the ability of natural environments (or images of natural environments) to mitigate cognitive load, discounting, and impulsivity in a contemporary environment—where these forces, along with screen time, advertizing and other marketing forces can drive unhealthy dietary choices [70]—is an obvious area of consideration. Personal experience with the growth of edible plants (through residential-, community-, and/or school-based gardening) and the subsequent promotion of healthy dietary choices is yet another [71,72]. However, one malleable dimension of personality—conscientiousness—may also represent a more specific point of intersection.

It is now widely recognized that personality trait changes are commonplace in adulthood; conscientiousness is one of the major dimensions of personality that can undergo remarkable change in the period between adolescence and midlife [73]. Increases in conscientiousness during adulthood predict improved mental and physical health over time [74]. Moreover, increases in conscientiousness during the influential ages of 13–21 appear to place young adults on a trajectory toward higher prosocial behaviors later on [75]. Individuals scoring high on conscientiousness appraise natural environments as highly relevant for their emotional well-being [76]. They are also more likely to choose healthy foods [77,78].

The available evidence provides a sound argument that engaging in healthy behaviors can *increase* conscientiousness [79]; in turn, the rewards associated with a specific lifestyle habit, e.g., physical activity or cognitive restoration in a natural environment, may motivate an individual to broaden the scope of behaviors associated with conscientiousness. This speculation will require research validation; however, researchers are beginning to track the ways in which day-to-day positive affect, feelings of engagement, purpose, and meaning in life, as well as curiosity and creativity, interact with dietary choices in the short and long term [80,81].

Of course, there is also the obvious dilemma related to the ways in which income-driven increases in global food demand are connected to environmental degradation and biodiversity loss. The consequences of clearing of savannas, grasslands, and tropical forests to accommodate a palate that is increasingly fond of Westernized fare require urgent research attention. If certain dietary patterns are connected to the promotion of mental health, how would such diets differ in their impact on environmental degradation?

At least one landmark study has initiated much-needed dialogue [82]. If urbanization and income increases continue on their current trajectory, by 2050, the global shift in dietary pattern will be as follows: 15% more calories, 11% more protein, 61% more calories in non-nutritive foods and beverages, 23% more pork and poultry, and 31% more beef and ruminant meat, 58% more dairy/eggs, and a staggering 82% more fish and seafood. On the other hand, there would be an 18% decrease in servings of fruits and vegetables and a 2.7% decline in plant protein intake. Moreover, there would be a 32% increase in dietary-driven greenhouse gas emissions.

However, in comparison to projections of the current global average diet, the global adoption of more traditional dietary patterns through 2050 painted a different picture in health outcomes and the environment. Using an average of three modeled traditional diets—the Mediterranean diet, a vegetarian diet, and a pescetarian diet (vegetarian diet that allows fish/seafood)—there would be a 43% reduction in GHG emissions compared to the current standard global average diet. In addition, an even combination of these three more traditional diets would require, on average, 540 million hectares *less* in land demand vs. the current standard global average. The modeling showed, unsurprisingly, that there would be a reduction in major non-communicable disease (NCD) risk accompanied by a transition to more traditional diets [82].

It should be pointed out that the Mediterranean diet is a broad term. From an ancestral perspective, there are many regional and local differences in food availability, as well as culturally determined influences on components such as meat content [83]. Within Italy, research suggests that high adherence to a Mediterranean-style diet equates to about 30% less meat intake vs. adults who maintain a low adherence to the traditional pattern [84]. Although the 2050 projection research above does not highlight diets that are the *most* effective at promoting mental health, nor are the three dietary patterns called out by the researchers necessarily the *most* sustainable, it provides a home base to begin complex discussions. How to feed a global population quality nutrition that promotes health, lowers NCD risk, and places the least amount of burden on natural environments?

## Microbial ecology

“Washing May Be Harmful, Kids” *Milwaukee Journal*, Wednesday January 17th, 1973: report from the Annual Meeting of the Milwaukee Academy of Medicine [85], page 1.

He [Dr René Dubos] suggested that overzealous sanitary efforts, a “*lack of contact with the thousands of usually innocuous microbes in the outside environment*,” might compromise the normal development of the immune system. He argued that for normal development and protection throughout life, to build up tolerance to harmful agents, a child’s environment be dirtied up in a controlled manner. Furthermore, he said that a lack of contact with the thousands of usually innocuous microbes in the outside environment at birth could retard the development of the newborn’s defense mechanisms. Dubos acknowledged that his suggestions were not in line with contemporary thinking and that he “*probably would not live long enough to find out whether he was right*.”

page 7 “Therefore, he suggested that science should decide to expose the young to a wide variety of the ever-present microbes to build up tolerance….”

In 1973, Dubos floated a bold suggestion during his keynote address to the Milwaukee Academy of Medicine. He cautioned, presumably to the astonishment of many in the audience, that North American children were being over-sanitized, and an associated loss of tolerance was not without consequence. As stated in the newspaper report cited above, his contention was certainly not in line with any mainstream thought *circa* early 1970s. This was the dawning of the era of the sanitizing-product industry (antimicrobial consumer goods were already a 1-billion USD industry in 1966 [86]).

His address was 16 years before the publication of the so-called “hygiene hypothesis,” which suggested that the global rise in allergic disease could be related to diminished opportunity for early-life exposure to pathogenic microbe exposure via increased hygiene, antibiotics, and smaller family sizes [87]. It was decades before Swedish physician Agnes Wold proposed a more general gut microbial deprivation hypothesis, one that included “*low exposure to bacteria via food or the environment in general. All this results in an* ‘*abnormally*’ *stable microflora*” in Westernized nations [88]. It was decades before the immune-modulating properties of non-pathogenic microbes would be scientifically postulated to be of value in neuro-emotional health (a proposition in which Dubos was referenced) [89].

Scientific consideration of exposing the young to a wide variety of bacteria, as Dubos proposed, is essentially the cornerstone of ongoing research under the “biodiversity hypothesis” realm. The biodiversity hypothesis has combined the more narrow hygiene and gut microbial deprivation hypotheses into a wider scope that includes the crisis of global biodiversity loss; the broad view includes the ways in which diminished contact with natural environments and loss of total biodiversity (including microbes) intersects with human health [90].

Co-evolutionary discussions of microbes and humans, so common today, are also an extension of what Dubos had proposed. He is credited with this, not by the present authors, but by his own peers writing in the *New England Journal of Medicine* (1973)—“*The term autochthonous has been applied to certain constituents of the flora by Dubos to indicate that the recent evolutionary development of these bacteria has been in concert with that of the host*.” Further, citing the work of Dubos, it was queried whether “*the intestinal microflora might be regarded as an organ of the body, the proper function of which, like that of other organs, might be related to optimal health*?” [91]. If evolutionary co-development brought about the existence of an organ (microbiota), and that organ interacts with other organs, the brain would surely not be excluded from the mix.

The concerns of Dubos regarding the increasingly sanitized world, although speculative, were not based on mere opinion. Despite becoming a scientific generalist, he was first and foremost a microbiologist. He began his career by discovering an antibiotic; however, he made it clear early on that it was a potentially dangerous practice to tinker with microbial ecology. In his landmark, original research paper with physician Russell Schaedler, entitled “The Digestive Tract as an Ecosystem” (1964), they quote

“*Thus, the composition of the microbial flora, which depends upon environmental and internal factors, in turn determines the kind of biologically active substances which are released from the digestive tract into general circulation…the different parts of the digestive tract, the microorganisms which it harbors, and the conditions which govern the interplay between these various components, thus constitute a highly integrated ecosystem. Any change in any one of these components is likely to affect the equilibrium and functions of the system as a whole, and thus to have generalized physiological effects*” [92].

It is very unlikely that Dubos would be surprised with the recent discoveries indicating that microbes extend a massive reach: from atmospheric chemistry and cloud formation [93,94], to mate selection and the remarkable ways in which plants and soil microbes interact to determine growth characteristics and even insect feeding behaviors upon the plant, microbes matter [95-97]. Had he lived long enough to see the dismantling of common myths, such as those which presumed bodily compartments such as the female womb during pregnancy is sterile [98], that the vascular endothelium and subepidermal regions are sterile in healthy adults, or that soil/water-derived microbes cannot be found in the brain in the absence of significant immunosupression and/or tissue destruction [99,100], he probably would not be surprised. Although the microbial contribution to general ecology has been historically and grossly overlooked [101], Dubos was cognizant of the connection:

“*In terms of the total economy of nature, the creative associations in which microbes are involved are probably far more important than are the diseases that they cause, or than the practical uses that man makes of them. Indeed, the entirely new creative processes that these associations represent give to the phenomenon of symbiosis a significance which transcends analytical biology and reaches into the very philosophy of life*” [102].

and

“*Many other protective mechanisms involving the participation of the normal microbes of the body will certainly come to light as attention is focused on the problem.*”

“*The difficulties that may follow antibacterial therapy are in fact similar in essence to those encountered in any attempt to control predators in nature*.. *Whether the method of treatment affects animal predators in the wilderness or the bacteria in the gut, it is always risky to tamper with the natural balance of forces in nature*” [103].

Throughout the 1960s, Dubos was involved in a series of studies related to DOHaD, including those with germ- or specific-pathogen-free animals housed in sanitary conditions. He also accumulated detailed analysis of the ways in which the normal intestinal microbiota could be disturbed and associated the changes to various aspects of health. In addition, Dubos and colleagues discovered ground-breaking interactions between nutrition, psychological stress, infections, and the intestinal microbiota [104-109]. In a summary that might seem lifted from a recent newsworthy scientific publication *circa* 2014, Dubos summarized some of his work on these intestinal microbiota interactions half a century ago—

“*The implications of these findings are many. For example, they compel a reinterpretation of the meaning of nutritional requirements of animals and men; they also raise new problems with regard to factors which condition resistance to microbial disease. It is clear that many characteristics assumed to be inherent in an individual can in reality be determined by the microbial flora of the intestinal tract*” [110].

Human research has recently made connections between greater diversity of intestinal microbiota, lowered markers of inflammation, and greater adherence to traditional dietary patterns [111]. Human research has connected predominant bacterial phyla in the gut with epigenetic regulation [112]. International studies have linked urban residence with higher levels of inflammatory markers such as C-reactive protein among adults [113-115]. There are likely many undetermined ways in which psychosocial and environmental stressors, some that may be unique to urban environments, can explain these connections [116]. Interestingly, the early origins of later-life inflammation have been the subject of some research, with results indicating that exposure to microbial diversity during infancy is associated with lower CRP in adulthood [117].

The use of 16 s rRNA sequencing techniques has addressed many of the limitations of stool culture methods. Researchers have found that, at least in Westernized urban dwellers, the slow differentiation of gut microbial diversity relative to our non-human ancestors—one that occurred over millions of years—appears to be undergoing a dramatic acceleration [118]. The advances in laboratory technique have shown that diet is a player in gut microbial diversity and that ancestral diets are more aligned with an intestinal microbiota profile distinct from that linked to chronic illness [119]. The examination of well-preserved ancient human coprolites (also known as paleo-stool) shows that the microbiota profile reflects ancestral dietary practices and culture, and its broad composition more closely resembles that of a modern hunter-gatherer vs. a Westernized urban resident [120,121].

Unsurprisingly, hunter-gatherers and other rural dwellers in Africa and South America have higher levels of microbial richness and diversity [122-125]. Approximately 35% of all lactic acid bacteria isolated from raw fruits and vegetables survive gastric conditions, and the diversity and functionality of microbiota of traditional/indigenous foods and beverages is far more complex than previously realized [126,127]. Remarkably, the gut microbiome appears to co-evolve with regional dietary patterns (and specific foods within them) via horizontal gene transfer from extrinsic microbes that allow for the breakdown of novel carbohydrates [128]. Further still, there are uncanny functional resemblances between plant root and human intestinal microbiota that may be of evolutionary significance [129].

In order to exert their far-reaching physiological effects, beneficial microbes need not become part of the established residential intestinal microbiota—nor do they have to be live. For example, in a recent experimental study, intake of a heat-killed strain of *Lactobacillus brevis* had a beneficial influence on circadian locomotion and sleep rhythms [130]. With emerging research showing that mere fragments of microbes (e.g., microbial DNA vs. live viable bacteria) can signal pathways, including the production of inflammatory-mediating cytokines [131-133], it is clear that we are merely scratching the surface of functionality.

As mentioned in the Part I introduction, the combination of Westernized dietary patterns and increasing microbial sanitization (antibiotic/antimicrobial product use) may play a synergistic role in relation to marked increases in allergic and autoimmune conditions in developed nations [134-136]. For further discussion on these relationships, and what has been described as a global epidemic of allergy, the reader is referred to detailed and well-written reviews [137,138]. The implications of this detrimental synergy are not restricted to allergy and autoimmunity [35].

Antibiotic-induced changes in gut microbiota seem to place a higher burden of oxidative stress on an animal [139]. Researchers are also connecting gut microbial diversity (or loss thereof), antibiotics, and antimicrobial agents (e.g., triclosan) with obesity [140,141]. Experimental studies show that early-life low-dose antibiotic induces metabolic alterations and influences the expression of genes involved in immunity. Low-dose antibiotic also enhances the effect of high-fat diet induced obesity. These changes are transferrable to germ-free animals when low-dose antibiotic-associated microbiota are transplanted, indicating a causal role for dysbiosis [142].

The critical point in the preceding research is that these results are noted with *low-dose* and subtherapeutic antibiotics, which raises multiple questions concerning broad environmental sources of antimicrobials. Residual levels of antimicrobials in the global food supply are now a significant concern to researchers [143-145]. Strong marketing forces push the commercially lucrative, yet very false notion, that our bodies and the surfaces with which we make contact must be routinely cleansed of all microbial life [146]. Given that 45% or more of neonates are exposed to antibiotics in North America [147], and emerging links between this exposure in infancy and subsequent overweight status through childhood [148,149], the urgency with which this topic needs to be addressed is now obvious.

Other remarkable diet-microbe connections are emerging. The rapid appearance of ultra-processed, ready-to-eat foods may be increasing our intake of advanced glycation end (AGE) products within those foods [150]. AGE are highly oxidant compounds formed through the non-enzymatic reaction between reducing sugars and free amino acids and are much higher in foods prepared with high temperature in the absence of water. AGE have been shown to reduce the growth of beneficial microbes in human and animal samples [151]. Higher intakes of dietary AGE are associated with a decline in cognitive function [152].

Agents such as non-nutritive sweeteners have been shown to inhibit the growth of bacteria, diminish intestinal microbial diversity, and encourage fat accumulation and glucose intolerance in experimental studies [153-156]. Environmental pollutants in air and food have also been shown to cause significant changes in gut microbiota [157,158]. On the other hand, the ability of select non-pathogenic microbes to assist in the elimination of environmental toxins from the body of mammals is becoming more obvious [159,160]. These findings, as well as others related to allergy and obesity research, should not stand alone. They are clearly intertwined with other silos, including broad discussions of mental health and the emerging research surrounding a microbiome and mental health connection [161].

## Hygiene, allergy, and mental health

In 1972, Dubos reported that when female specific-pathogen-free mice are fed low-quality protein during the perinatal period, the brain content of dopamine and norepinephrine in offspring is diminished [162]. In 1986, Linda Hegstrand and colleague R. Jean Hine reported a remarkable discovery to the scientific community—compared to germ-free animals, conventionally raised animals have higher levels of histamine in the hypothalamus [163]. While best known for its connection to allergy, brain histamine in general, and hypothalamic histamine in particular, is a key factor in arousal and behavior [164]. Perhaps underappreciated at the time, their work was a milestone in the gut microbiota-to-brain axis.

Although the hygiene hypothesis itself remained focused in the realm of allergy, it was proposed in 2002 that the ways in which beneficial bacteria influence T-helper cells (TH1:TH2 balance) in allergies might also extend to conditions where neurocognitive symptoms are common. When this seemingly outlandish proposal was made (accepted Aug, 2002), it was pointed out that allergies are a frequent overlap with chronic conditions in which emotional and somatic symptoms are a central feature. If TH1:TH2 imbalance was a driver of many emotional and somatic symptoms, then a mitigating role for beneficial microbes was theoretically possible [89].

As the traditional focus on harmful microbes began to shift toward lactic acid bacterium and commensals, novel scientific frameworks began to bridge the immune/nervous systems to neuropsychiatric health via non-pathogenic microbes and nutrition. Although in the early 2000s, these discussions were forced to the fringes, far removed from the safety net of the impending microbiome frenzy, Dubos was not overlooked in the reference lists [89,165]. Over the last decade, experimental and preliminary clinical studies have produced findings that would have been virtually inconceivable to most at the end of the 20th century. Strangely, Dubos has an appallingly low number of citations (to what should have been milestone papers—e.g., [162]) on Google Scholar.

Concerning his own groundbreaking studies on the microbiota of the total environment and what it might mean to human well-being, Dubos referred to himself as simply walking in Metchnikoff’s footsteps [166]. Within his book *Man Adapting* (Yale University Press, 1965), he details his own experiments on fecal microbiota transplantation and immunity. He found that a more sanitized environment reduced intestinal microbial diversity in mice. In this remarkable text, he makes it clear that differences in ancestral evolutionary development are reflected in the presence or absence of members within the indigenous microbiota of various species. As usual, he demonstrated his awareness of socio-ecological interplay in humans:

“*Whatever the exact mechanism of the interplay between nutritional state and the microbiota of the digestive tract, it is clear that rate of growth, nutritional requirements, and efficiency in food utilization are characteristics influenced by* [commensal bacteria]…*But sanitary practices and other aspects of the ways of life may also play an important part by affecting the indigenous microbiota…histological differences observed in the intestinal mucosa depending upon the socioeconomical status may be relevant to the problem, since they probably reflect the intensity of the inflammatory response to the so-called* ‘*normal*’ *flora*.” [167].

Fast-forward and studies using germ- and pathogen-free mice have shown definitively that microbes can influence brain and behavior [168,169]. In one of the most remarkable experimental studies to date, the transplantation of fecal microbiota from donor mice raised on a high-fat diet into lean mice (who had been raised on a normal chow diet) resulted in altered neurologic function. Specifically, there were changes in behavior suggestive of anxiety, increased stereotypical behavior and decreased memory [170].

These studies show us that microbes matter; however, there are massive limitations when using rodent studies as a means to provide translation in general [171] and especially toward complex issues such as mental health [172-174]. How far can we carry the clinical relevancy of germ-free rodents and/or elevated plus mazes used to measure rodent “anxiety” vis-à-vis the equally complex human ecosystem? Animals in the natural environment might experience dysbiosis—but they are not living la vida germ-free. Although Dubos put DOHaD on the map with his own germ- and pathogen-free rodent studies 50 years ago, he would almost certainly agree with a critical note concerning global mental health—psychiatry is not applied neuroscience [175].

This sometimes inconvenient yet perspective-providing fact can, at times, be easily overlooked. While many future promissory notes are now being signed off with the signature of “microbiome,” Dubos warned about select overselling of similar technological advances in his time. He referred to their promotion at scientific meetings as a combination of “intellectual dishonesty” and “intellectual escapism” when they ignore glaring holistic environmental variables that are not nearly as press-release-worthy [176]. In the contemporary tweet-driven world of pseudo-medico social media [177,178] and overhyping of research by academics [179], his concerns may be particularly relevant.

Notwithstanding translation difficulties, the emerging rodent studies have provided some undeniably valuable clues to possible mechanistic pathways between microbes and brain function. Collectively, the experimental studies indicate that beneficial microbes might influence neurocognitive function, behavior, and perceived stress by various pathways: direct communication to the central nervous system (most likely via the vagus nerve), controlling the intestinal barrier (and limiting systemic low-grade inflammation), improvement of nutritional status (phytochemical absorption and neurotransmitter precursors), reducing systemic oxidative stress, regulation of glucose control, and limitation of uremic toxin production [180-183]. Undoubtedly, others will be revealed.

In the clinical setting, several studies have shown that oral probiotics, or fermented foods/beverages inclusive of beneficial bacteria, may provide value in end points such as depression, anger, anxiety, and daily mood [184-189]. Perhaps the most interesting study to date involved functional magnetic resonance imaging (fMRI) assessments before and after the 1-month consumption of a microbial-fermented vs. unfermented food—with imaging results suggesting a reduction in vigilance to negative environmental stimuli among the healthy adults that consumed the fermented dairy product [190]. Research has also identified intestinal microbiota differences in those with major depressive disorder and chronic fatigue [191,192]. On the other hand, numerous studies show that those with depressive symptoms and high levels of psychological distress engage in dietary patterns that are far removed from a healthy traditional pattern that might otherwise support microbial diversity [11].

## Beyond food and probiotics

While intestinal microbiota and dietary interactions have received considerable international attention, it is worth noting that lactic acid bacteria, bifidobacteria, and numerous other potentially beneficial microbes are found throughout natural environments. For example, lactic acid bacteria make up a significant portion of the phyllosphere (the aboveground botanical habitat for microbiota) even under extreme conditions. Moreover, bifidobacteria are found in the rhizosphere (narrow band of soil just under the surface, associated with root secretions) of some of the harshest environments on the planet Earth [193,194].

Indeed, significant differences have been noted between the microbial content of urban vs. rural air—results showing greater overall diversity in the rural near-surface atmosphere. There is also relatively higher abundance of bacteria in rural samples, including the microbial phyla that make up the portions of the human skin and intestinal microbiota such as *Actinobacteria*, *Bacteroidetes*, *Firmicutes*, and *Proteobacteria*. In particular, 16 s rRNA sequences assigned to the lactic acid bacterial group are higher in rural air [195]. A 4-day, 3-night trip to a forest setting has been associated with a significant reduction of serum levels of the TH2 chemokine MDC/CCL22 [196], which allows for speculation concerning microbial mechanisms of action.

The results of a recent European studies provide contextual meaning to the public health implications of biodiversity loss at the microbial level. Lower biodiversity of vegetation surrounding one’s residence has been associated with higher odds of an allergic IgE reaction to common allergens and lower diversity of select bacteria on the skin [197]. In fact, the extent to which green areas (within 2–5 km from primary residence) are inversely associated with atopic sensitization in children may be a product of the way these natural environments differentially shape commensal skin bacteria [198].

It seems likely that this is the type of research with which Dubos would have been most impressed. Reductionist technique immediately provides clear relevance to the broad environment. Since these environmentally influenced skin microbes, members of the *Gammaproteobacteria* family, in particular, can influence the immune system beyond the skin itself [199], similar relationships may be found in connection to mental health.

## Outdoor activity and microbiota

A number of academic articles have concluded that modern humans would do well to exercise in ways that reflect our hunting and gathering past [200,201]. Indeed, physical activity in natural environments may increase a sense of vitality and motivate an individual to adhere to future exercise routines [202,203]. This is of particular importance in those with mental health disorders or obesity in whom motivation is a primary barrier to physical activity [204]. Common symptoms of mental disorder are predictive of subsequent time spent sitting later in life [205]. Exercise feels differently for those who embrace it as part of their lifestyle vs. individuals with depressive symptoms or obesity—the latter groups report lower pleasure ratings and post-exercise energy levels, while during the activity perceived exertion levels are higher [206,207]. Since fostering positive emotions in association with the experience of exercise can alter the way exercise feels and encourage future participation [208,209], the implications for the value of natural environments are obvious.

Beyond the classically known benefits of physical activity (cardiovascular, respiratory function, etc.), and the more indirect sensory and psychological (mood, cognitive restoration, etc.) value, there is a third dimension of potential benefit related to outdoor activities. We consider the potential diversity of microbial contact to be important and often overlooked consideration. In addition to vegetation as mentioned above, outdoor air has a greater phylogenetic diversity of bacterial communities than does indoor air [210].

Outdoor play, gardening, or any activity that might bring contact with soil, trees, and plant life will increase the opportunity for exposure to microbial diversity. If the activity is conducted in natural environments, it increases the likelihood that the individual will inhale natural phytoncides (aromatic chemicals secreted from trees and ornamental plants) that are associated with human stress reduction and immune system benefits [211,212]. These airborne phytoncides, found at higher levels in natural (vs. urban built) environments, are a product of interactions between phyllospheric microbiota and the plant; for example, the application of antimicrobials to the aboveground portions of flowers leads to a halt in linalool emissions [213], a chemical with known mood-regulating properties [214].

Recently, it was reported that athletes who make frequent contact with natural grass, soil, and mud (Rugby players) have greater diversity of gut microbiota vs. physical size, age- and gender-matched controls [215]. The fact that Rugby players engage in a unique form of exercise that brings them in direct contact with the Earth was not mentioned by the authors—the conclusion was focused primarily on differences in exercise intensity. However, intestinal microbiota is not solely a product of diet, and the influence of non-diet-associated terrestrial microbes on intestinal and systemic physiology is an open question [216-218]. Undetermined environmental factors that are unique to geographically distinct human societies may even transcend the dietary influence on gut microbiota composition [219].

The administration of live *Mycobacterium vaccae* has recently been shown to improve cognition and reduce experimental signs of anxiety in an animal model [220]. *M. vaccae* is a generally non-pathogenic microbe that, depending on region, can be found in varying amounts within soil, mud, and/or natural water wells [221]. An earlier 2004 study involving an injection of *M. vaccae* (non-placebo-controlled) in patients with lung cancer indicated that it may attenuate some aspects of deterioration in quality of life [222]. Media positioning of *M. vaccae* as the “happy” widely available soil bacterium is common, and it may lose sight of the fact that this particular microbe is associated with intellectual property and a long list of patents.

*M. vaccae* is often used as a broad term that does not address its many specific strains. There has been little discussion surrounding the specificity of *M. vaccae* strains and emotional health; probiotic studies in the gut-brain realm suggest that not all microbial strains are alike [183,223,224]. Are there strain-specific effects of *M. vaccae* in neurocognition? If so, in what environments are these strains most abundant? In our context of natural environments, we would be interested to see if there are behavioral changes as a result of other non-commercially linked, non-pathogenic soil, or phyllosphere-associated microbes.

## The total environment

“*I cannot help but expressing my belief that living things, including man, respond not only to heat, humidity, light and other obvious climatic components which are readily perceived by the senses, but also to many other environmental factors not readily identified, and in part, still unknown*” [225].

Human biometeorology is an interdisciplinary science concerned with interactions between atmospheric processes and living organisms. In the context of climate change and environmental degradation, this field has experienced renewed interest [226]. Natural environments can also be distinguished from the urban built environment by some of these atmospheric differences. For example, trees may help mitigate the thermal discomfort associated with urban heat island effects [227]. On the other hand, urban warming may encourage herbivorous pest abundance and tree destruction [228].

One of the more controversial aspects of meteorological conditions as they relate to health of living organisms is that concerning the relative content of charged air ions. Dubos maintained an open mind on the topic. Despite suggesting that air ions may have untold benefits, including an ability to modify beneficial microbes within the intestinal tract [167], page 124], Dubos took exception to any association between him and commercial ion-generating devices [229]. Unquestionably, the research related to air ions has been the subject of pseudo-medical exploitation in marketing claims associated with ion-generating devices.

However, since his writings on the topic, emerging research is undoing the notion that air ions are without *any* relevance to the growth and function of living organisms. The preponderance of available evidence shows that air with a relative increase in negatively charged ions may help facilitate positive mental outlook [230-232]. The relative concentration of negatively charged air ions is higher in natural environments [233-235] and lower in urban (as well as indoor) environments that are subject to airborne pollutants [236,237]. Forests and areas rich in vegetation can help offset the loss of negatively charged air ions within urban environments [238]. Remarkably, negative air ions also appear to increase phytoncide release from trees [239], indicating the potential relationship to some of our previous discussions.

In daily life and sleep, our ancestors would have made much more frequent direct contact with the Earth. Some researchers have speculated that less frequent contact with the surface of the Earth may translate to a deprivation in contact with abundant surface electrons, which in turn could impair diurnal electrical rhythms and burden the antioxidant defense system [240]. Only properly designed studies can determine whether this particular speculation may have broad health implications.

Finally, a less speculative area of total environment research is one touched upon in Part I—that of light. As mentioned previously, our current relationship with light is divergent from our ancestral past—higher levels of light at night (LAN) and lower exposure to light during the day are now commonplace. Here, we briefly mention the importance of the blue portion of the light spectrum. In a recent North American study, it was reported that the daily exposure to light intensity above 1,000 lux (levels associated with outdoor light) was only about an hour on average. Interestingly, in that study, those who accumulated the majority of their intense daily light exposure (levels beyond a 500-lux threshold) in the early part of the day were more likely to have a lower body mass index (BMI) [241]. Why would getting outdoors in early morning light make a difference?

One possibility is the higher amounts of shorter wave blue light in the early part of the day [242]. Given the emerging research on appropriately timed blue light exposure and positive mental health [243], it appears to be yet another feature of the total modern environment that we may be missing, at least in the daytime. While our standard interior lighting cannot compensate for the amount of blue light found in natural daylight [244], only a small amount of evening blue light can suppress melatonin. The consequences of evening-night use of modern interior lighting and multimedia screens (i.e., higher blue light) may be compromised sleep, fatigue, mood, and metabolic disturbances [245-247].

Circadian disturbances have been shown to induce dysbiosis in both mice and humans—and promote glucose intolerance and obesity that are transferrable to germ-free mice upon fecal transplantation [248]. Therefore, we have much to learn about the far-reaching effects of light and its components. Dubos would probably have been less interested in the clinical utility of various doses of melatonin to mediate jet lag and more interested in its evolutionary origins [249] and the lifestyle drivers [250] behind why its use as a supplement has dramatically increased in developed nations [251].

## Heterogeneity of cities, vulnerable populations

After weaving our way through each of our main sections—natural environments, nutrition, and microbes—we must underscore that not all global cities and districts within cities can be painted with the same brush [252]. On a global scale, however, environmental injustices are common and the concentration of urban vegetation and biodiversity is often slanted in favor of the affluent and less vulnerable [253-256]. Any slant away from natural environments is not a zero-sum game. It forces a question: what might be there in its stead?

Lack of green space in urban environments, especially in socioeconomically disadvantaged areas, is often coincident with more grey spaces. These are residential areas with disproportionate industrial and commercial activity, heavy traffic, bars, liquor stores, convenience stores, and fast-food outlets [257-263]. Although these zones may contain limited vegetation, there is an abundance of visual marketing of unhealthy lifestyle choices—e.g., fast food and tobacco [260-262].

Therefore, many of the mental health benefits of green space may be explained top-line by the *absence* of grey space—i.e. *less* noise, traffic, walkability, environmental toxins, and visible marketing that otherwise cajoles residents toward (and less actual opportunity to partake in) unhealthy lifestyle choices [11,264,265]. The financial strain and fatigue associated with psychological distress in socioeconomically disadvantaged areas enhances the attractiveness of cost and convenience in low-nutrient food choices [11]. Perhaps time spent indoors may not be completely disadvantageous if the external environment is polluted and dangerous.

Moreover, we must acknowledge that generalized stress (and specific stressors—e.g., acoustic stress), pollution, marketing toward junk food, smoking, antibiotic prescriptions [266-270], and other socioeconomically slanted environmental forces described above might continuously influence microbial diversity and dysbiosis [11,157,158,271-273] unless they are addressed. This puts a dampener on the oft-unchecked enthusiasm with which single (or a few) strains of isolated bacteria are heralded as a mental health solution. It is hard not to draw parallels between the microbiome euphoria of today, and the emergent technological research Dubos addressed in 1969:

“*This is wonderfully entertaining, titillating kind of science fiction. We organize meetings about it in all sorts of pleasant places to talk about this, and that saves us the responsibility of walking across the street, where 100,000 children are being poisoned every day by lead in paint…something can be done immediately about this problem, but it is not being done because it is not of sufficient interest or as exciting intellectually*” [176].

Put simply, are we to imagine a world in which the socioeconomically disadvantaged line up for laboratory-generated microbial products, while the environmental forces that might allow dysbiosis to be the default mode remain in place? Are they to be sprinkled on the heavy metal-contaminated grounds [274] of the less accessible, inadequately maintained green areas often afforded to the vulnerable [275,276]? These absurd notions could be inferred when the complex circumstances that contribute to mental ill-health (or positive emotional well-being) are ignored, and furthermore, when it is assumed that the at-risk are among those who can find the means to secure expensive, commercially driven products that are touted at meetings in pleasant places. If animal studies involving diet-microbiota-obesity interactions are an indication [277], fecal transplant in mental health will be limited if the environmental forces are pushing a separate and continuous reset button labeled “dysbiosis.” If there is an end-game to the potential benefits of microbial administration in positive mental health, it is much more likely to be derived from ecological vs. purely pharmacological and commercially driven considerations.

Having read stacks of Dubos writings, we found one paper [278]—*Science and man’s nature. Daedalus: J Am Acad Arts Sci 1965, 94:223–244*—that captured so many of his views in a single place. The following extended quotes from this classic piece are presented with permission of MIT Press.

“*The immediate effects of the conflict between the Paleolithic constitution of man and the exigencies of modern life can be documented by chemical, physiological, and psychological measurements, but little is known of their long-range consequences. There is no doubt, however, that many physiological disturbances have their origin in the conflict between the modern environment and the Paleolithic ordering of physiological functions*.”

“*For many thousand years, man has modified his environment by using fire, farming the land, building houses, opening roads, and even controlling his reproduction. The all important difference, however, is that many modern applications of science have nothing to do with human biological needs and aim only at creating new demands, even though these be inimical to health, to happiness, or to the aspirations of mankind*.”

“*A more disturbing aspect of modern science is that the specialist himself commonly loses contact with the aspect of reality which was his primary concern, whether it was matter, life or man*…*one of the strangest assumptions of present day biology is that knowledge of living man will automatically follow from so-called* ‘*fundamental*’ *studies of the elementary structures and reactions of fragments derived from living things. In reality, a very different kind of knowledge is needed to understand the nature of the cohesive forces which maintain man in an integrated state, physically, psychologically, and socially, and enable him to relate successfully to his environment*.”

“*Science and the technologies derived from it now often function as forces independent of human goals. In many cases, as we have seen, knowledge creates concepts that man cannot restate in terms of his experience; and increasingly, technology creates services and products that man does not really need. All too often, knowledge and technology pursue a course which is not guided by pre-determined social philosophy*.”

“*Even though dangers are also inherent in the knowledge concerning automation, synthetic chemicals, or almost any other new technology, surprisingly little is done to evaluate the possible social consequences of these innovations*.”

“*The study of man as an integrated unit, and of the ecosystems in which he functions, is grossly neglected because it is not in the tradition which has dominated experimental science since the 17th Century. Such a study would demand an intellectual approach, as well as research techniques and facilities, different from those which are fashionable and professionally profitable in the academic establishment*.”

## Summary and future directions

René Dubos was a city-loving, technology-embracing microbiologist who was well versed in the value of reductionist technique. He readily acknowledged that ancestral experiences were often brutish and that the solution to contemporary problems was not to be found in a romanticized “return to nature” [103,279]. He understood that humans continue to evolve and never wavered from his optimism that the human evolutionary forward-march—with its cultural-genetic interactions and microevolutionary aspects that are now better understood [280,281]—was away from its brutish beginnings and toward a better place. Like some of our most forward-thinking public health officials of today [282], he recognized the complexity of his proposals. On the other hand, he noted that life may not be as miserable and brutish (as otherwise portrayed) if we were absent some of the technology currently delivered to us at a premium cost to the planet [283].

Amidst the optimism, he raised warning flags concerning unintended consequences. He placed them along a river-like course into which the tributaries of natural environments, biodiversity, microbial ecology, and quality of nutrition would flow. The ultimate destination of this collective current was toward generalized aspects of well-being, quality of life, and the promotion of personal and planetary health. He referred to this as humanistic biology and reminded his audiences that “*even when man has become an urbane city dweller, the Paleolithic bull that survives in his inner self still paws the earth*” [284].

A contemporary look at the work of Dubos would quickly reveal words such as these delivered to the World Health Organization’s 1969 Annual Assembly

“*Air, water, soil, fire and the natural rhythms and diversity of living species are important not only as chemical combinations, physical forces, or biological phenomena but also because it is under their influence that human life has been fashioned. They have created in man deep-rooted needs that will not change in any near future”* [285].

In 1968, while addressing an audience at the United States National Institute of Mental Health, he suggested that shifting dietary practices in affluent nations would take its toll in the mental health realm: “*It would be surprising if such acquired dietary habits, in addition to being physiologically objectionable, did not also have unfavorable behavioral manifestations*.” Pushing it further, he stated that humans, should they continue to become disconnected from nature in the emerging technological society, would begin to resemble animals in captivity: “*The domesticated farm animals and the laboratory rodents on controlled nutritional regimens in controlled environments will then become true models for the study of man*.” [286], page 67].

That highly controlled, sanitized, and calorie-dense environment, we are now learning, *is* associated with the promotion of inflammation and chronic disease development [35]. Dubos was also concerned with the industrial or public policy application of technological endeavors made without consideration of unintended consequences. As a way to exemplify his concerns related to unbridled enthusiasm toward technology, he often referred to the primary theme of the 1933 World’s Fair—Science Finds, Industry Applies, Man Conforms. Here, we provide a primary quote from that 1933 Guidebook:

“*Science discovers, genius invents, industry applies, and man adapts himself to, or is molded by, new things…Man uses and it affects his environment, changes his whole habit of thought and of living. Individuals, groups, entire races of men fall into step with the slow or swift movement of the march of science and industry*” [287].

Where does the swift portion of the march of science and industry meet with unintended consequences? For Dubos, the implications of such words within the *Guidebook* were obvious. His interpretation: “*It implied that man must conform to the environment created by industry, instead of using science and technology to develop conditions really suited to fundamental human needs…it is not man which must conform to technology, but technology which must be made to conform to the human condition*.” [288].

Decades before terms such as “biophilia,” “hygiene hypothesis,” “developmental origins of health and disease,” “green space,” “blue space,” “nutritional psychiatry,” “narcissism epidemic/empathy drought,” “intestinal microbiota ecology,” “microbial deprivation,” “fecal microbiota transplantation,” “epigenetics,” “biodiversity hypothesis,” “screen time,” “evolutionary mismatch,” et al. became popularized, Dubos was already directly (or indirectly by sewing together various strands of ideas and publications of his peers) researching, writing, and paving the way for these realms. For any researchers tempted to claim that trans-generational and/or social-behavioral interactions with microbiota and physiological end points are novel concepts, a read at his work on specific pathogen-free mice [167,289] would be a good starting point.

His concerns about the future implications of these broad issues were sometimes based on published evidence, and at other times, they were self-admittedly of a speculative nature. Critics of the day pointed out that much of his positioning remained on the side of opinion rather than available evidence; however, they still understood his primary message. Renowned philosopher of science David L. Hull captured it in elegant fashion: “*Although such gene pools can change over long stretches of time, the needs of humankind are so pressing that we can hardly pin our hopes on changes in our genetic endowment. If we are to survive, Dubos argues, we must exploit the plasticity of expression of our genetic endowment*” [290].

Based on research outlined in this review, his forewarnings may have at least some contemporary scientific validation.

Dubos began his scientific career in the most reductionist way possible—combing through countless soil microbes to get to the species which would help him synthesize a clinically relevant antibiotic. At the end of his life, he continued to underscore the necessity of the reductionist approach in experimental science. However, he was keenly aware of its limitations as a means to inform clinical relevancy to humans in their total environment. For Dubos, it was the interactions and synergy between micro- and macro-environmental variables that required more study.

“*Like other biological sciences, medicine is a complex structure resting on several supports. If it were limited to the art of the medical practitioner and to the reductionist philosophy of the biochemist, it would be a two-legged structure and would soon collapse through lack of balance and adequate support*” [291].

Each of our three main sections—natural environments, nutrition, and microbes—are potentially intertwined. For example, lack of sensory experience (visual, auditory, olfactory, and tactile) in natural environments could influence affect, cognitive restoration, stress resiliency, and sleep quality. In an environment where cognitive load and screen technology use is often high, this could very well influence dietary choices [292]. These dietary choices may, in turn, influence motivation and mood state [11].

Lack of experience in natural environments, or certain dietary patterns, may also influence individual microbial diversity. This, in turn, may have many health consequences. Lack of natural light and a compromised ability to secure normal circadian rhythms can interact with all of the above [243,248,293-297]. Physical activity, unquestionably of value to human health and well-being, may be more pleasurable in outdoor natural environments, with diminished perceptions of exertion and an increase in the likelihood that an individual will be motivated to engage in further physical activity [298-302]. Exercise therefore becomes yet another variable intertwined with dietary choices, affect, intestinal microbiota [303,304], natural environments, and so on.

As surely as discussions concerning beneficial microbes and brain health were on the fringes in the early 2000s [89], the tide may also be turning in another marginalized conversation—the non-thermal biophysiological influences of electromagnetic radiation (EMR) [305-307]. The median of total radiofrequency electromagnetic fields in Austrian bedrooms has almost doubled between 2006–2012. Notably, analysis of all households showed higher loads (over 3× higher median) in urban than in rural areas [308]. What are the synergistic effects of EMR and environmental contaminants [309]? For now, we can only conclude that EMR exposure differs significantly from our ancestors; however, it would seem remarkable if an increasing human-generated EMR load had absolutely no consequences to life on Earth [310,311].

What are the psychological consequences of a *collective* deficiency of ancestral influences in the modern landscape? What is the fallout from more time indoors and *less* experience with variables associated with natural environments or parts thereof? The “less” we refer to is broad—it ranges from the diversity of birdsong and non-pathogenic microbes to the blue spectrum of daylight and varieties of vegetation around a residence. It includes less dietary diversity via loss of traditional foods and the diminishing phytochemicals within them. In the context of evolutionary experience, we consider these to be modern deficits.

**Figure 1:**
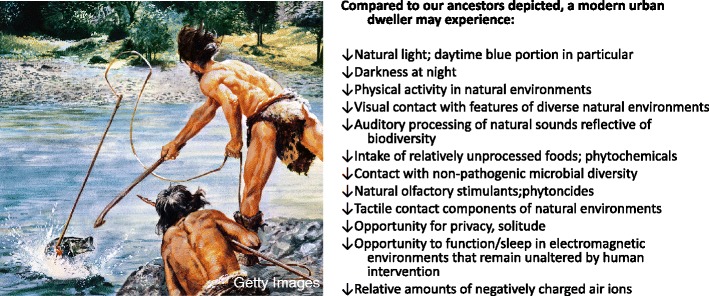
**Paleo-deficit disorder?**

We wonder could this *collective* deficit manifest in a “disorder,” a sort of paleo-deficit disorder, that while not pathological *per se*, taps into unrealized quality of life, empathy, perspective taking, low-grade anxiety, psychological distress, resiliency, and negative mental outlook? Could this deficit accelerate an individual toward the checkmarks required for medicalized diagnoses? Might the collective deficit in “Paleolithic experiences” compromise an individual’s ability to maintain optimal emotional health and by extension, prevent optimal health of neighborhoods, cities, societies, and nations, especially those undergoing rapid urbanization? Please see Figure 1. Our primary concerns and questions are not dissimilar to those of Dubos.

The evidence discussed in this review forces many questions concerning the deeper level essentiality of natural environments and the elements within them. Trees, of course, remove millions of tonnes of airborne pollutants from cities each year [312]. Their presence can therefore diminish some of the direct effects of grey space, while at the same time adding to the benefits of microbial diversity. Yet, they appear to provide benefit at a much deeper psychological level. Dubos raised this possibility, and others like it, to scientific colleagues and the public 50 years ago. His concerns related to the mental health consequences of environmental degradation are no less relevant today. On the contrary, we consider them to be urgent.

Future research in the area of environmental variables and mental health should consider the application of simple, validated instruments such as the Nature Relatedness (NR-6) [313] and scales of contentiousness [79]. For example, how might those who score high on nature relatedness differ in their dietary practices? Might dietary practices and frequency of “contact with biodiversity” influence their individual microbiome? How does a community-level nature relatedness interact with social capital and regional quality of life [314]? How does blue space influence urban mental health [315]? If mindfulness, contentiousness, and the ability to perceive nature’s beauty are essential components to achieving the maximal health benefits associated with natural environments [316-320], then it seems fair to ask if the technological society, as Dubos claimed, is compromising our ability to be mindful and capture those perceptions within such environments.

The existing research, despite its many limitations, does suggest that natural environments and “access” to biodiversity (including microbial diversity) are indeed essential to public health, especially in urban settings. We need to learn more concerning the socioeconomic and political factors that influence ecosystem services and promote healthy cities (or perceptions of a healthy city) for all citizens [253]. This entails input from minorities, immigrants, and the socially disadvantaged. As others have pointed out, in this process, we cannot assume that simply adding “green” will provide a simple solution [321,322].

At the same time, we cannot assume that in the Gold Rush known as the microbiome, where so many are staking a claim, microbes will provide us with all the answers. Lest our own very broad discussion get confined to the all-microbe *zeitgeist*, we should point out some important and seemingly overlooked references. Stress can promote systemic inflammation independently of microbiota [323]. Moreover, whatever its deficits may be, the germ-free mouse maintains microbe-independent communication via neurotransmitters and the odortypes that can govern social behavior, mating, and reproduction [324,325]. Therefore, properly nourished, capable-of-reproduction germ-free mice might also provide some much-needed perspective.

As cited above, microbiota are undeniably linked to many variables highlighted throughout our discussion; at a certain tipping point [326], bacteria, viruses, and living members of the human ecosystem may amplify or abbreviate other paleo-deficits. Our more immediate concern, as it was with Dubos, is the *collective* deficit—the total environment—in which dysbiotic microbiota is a part. Put simply, one might have in their procession the most ideal microbiota profile, the gold standard against which all future commercial fecal products will be measured, and still fall well short of optimal mental health.

Our stance does not dismiss the strong probability that lifetime microbial experiences, especially the early ones, could have long-lasting influences on *how* and even *why* an individual responds in a differential manner to visual, olfactory, and other sensory aspects of natural environments. Microbial interactions with genetic factors will likely be linked to Nature Relatedness, emotional well-being, and intestinal or skin microbiota. Indeed, that is our primary point—in common parlance, “it’s all connected”.

Reading Dubos, it would be tempting to state that there is nothing new under the sun. Maybe we have reached a point where theory after theory, hypothesis upon hypotheses, are all variants on the same theme. Perhaps discussions of natural environments, biodiversity, microbiota, nutrition, and mental health are often one in the same. We consider this to be an important consideration simply because it might help guide plausible solutions.

Decades before the global rise in allergic diseases were considered to be at epidemic proportions; Dubos addressed membership of the American Academy of Allergy at its annual meeting. We close on his words, communicated in 1969, as keynote speaker to his audience of allergists:

“*The etymological meaning of allergy is, of course,* ‘*altered reactivity*.’ *In this sense, all aspects of life can be considered as manifestations of allergy, since most anatomical and functional characterizes of man can be modified by the stimuli*—*physical, chemical, social*—*that impinge on him throughout life…developing counter technologies to correct new kinds of damage constantly being created by technological innovations is a policy of despair…we must try to imagine the kind of surroundings and of life we want, lest we end up with a jumble of technologies that will eventually smother body and soul*” [327].
